# Supplementary material for: Modulation of Dopamine Receptors on Osteoblasts as a Possible Therapeutic Strategy for Inducing Bone Formation in Arthritis
Source: Cells. 2022 May 11;11(10):1609. doi: 10.3390/cells11101609 (PMC9140103; doi:10.3390/cells11101609)
Supplement: Supplementary file 1 [file cells-11-01609-s001.zip › cells-1703531-supplementary.pdf]

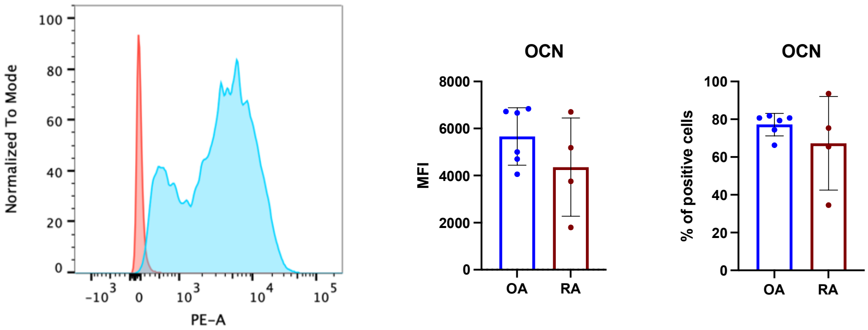

Supplementary Figure S1: *Osteoblast were stained for osteocalcin (OCN) by flow cytometry at passage 3. Left: representative histograms showing OCN expression in RA. Red: unstained control; blue: specific DR staining. Right: mean fluorescence intensity (MFI) and % of positive OCN stained cells in OA (blue bars) and RA (red bars). Histograms represent mean ± SEM. Each point represents one patient. OA n= 4, RA n = 5.*

Supplementary Table S1:
Antibodies used for IHC staining

Antigen	Clone	Company	Dilution 1/x
D1DR	rabbit polyclonal	Novus Biologicals	100
D2DR	rabbit polyclonal	LSBio	100
D3DR	rabbit polyclonal	OriGene	100
D4DR	rabbit polyclonal	OriGene	100
D5DR	rabbit polyclonal	LSBio	200

Supplementary Table S2:
Antibodies used for FACS analysis

Antigen	Clone	Fluorochrome	Company	Dilution 1/x
D1DR	L205G1	PE	Biolegend	50
D3DR	Polyclonal	Cy5	Bioss	100
D5DR	889022	AF405	R&D	100
D2DR	Polyclonal (rabbit)	/	LSBio	100
D4DR	Polyclonal (rabbit)	/	Bioss	100
Donkey anti-rabbit	Poly4064	PE	Biolegend	400
TH	Polyclonal (rabbit)	AF647	Bioss	200
Osteocalcin	R14-707	PE	BD Pharmingen	100

Supplementary Table S3:
Primers used for real-time PCR

Primer name	Supplier	Sequence
TRAP forward	Thermo Fisher Scientific	CACTTCAAGATCCCACAGAC
TRAP reverse	Thermo Fisher Scientific	AGTCATCTGAGTTGCCACAT
NFATc1 forward	Thermo Fisher Scientific	ATAACCAGTTTTTCCACGAT
NFATc1 reverse	Thermo Fisher Scientific	CACGAGGGGTCTCTGTAG
CTSK forward	Thermo Fisher Scientific	TTCCCGCAGTAATGACACCC
CTSK reverse	Thermo Fisher Scientific	GGAACCACACTGACCCTGAT
MMP9 forward	Thermo Fisher Scientific	GTAAGGAGTACTCGACCTGTACCAC
MMP9 reverse	Thermo Fisher Scientific	CCACTTCTTGTCGCTGTCAAAGTTCG
GAPDH forward	Thermo Fisher Scientific	AATCCCATCACCATCTTCCA
GAPDH reverse	Thermo Fisher Scientific	AAATGAGCCCCAGCCTTC
